# Mesenchymal Stem Cells Develop Tumor Tropism but Do Not Accelerate Breast Cancer Tumorigenesis in a Somatic Mouse Breast Cancer Model

**DOI:** 10.1371/journal.pone.0067895

**Published:** 2013-09-12

**Authors:** Lydia Usha, Geetha Rao, Kent Christopherson II, Xiulong Xu

**Affiliations:** 1 Division of Hematology/Oncology, Department of Medicine, Rush University Medical Center, Chicago, Illinois, United States of America; 2 Department of General Surgery, Rush University Medical Center, Chicago, Illinois, United States of America; Baylor College of Medicine, United States of America

## Abstract

The role of mesenchymal stem cells (MSCs) on breast cancer progression, growth and tumorigenesis remains controversial or unknown. In the present study, we investigated the role of MSCs on breast tumor induction and growth in a clinically relevant somatic breast cancer model. We first conducted *in vitro* studies and found that conditioned media (CM) of RCAS-Neu and RCAS-PyMT breast cancer cell lines and tumor cells themselves dramatically increased the proliferation and motility of MSCs and induced morphological changes of MSCs and differentiation into fibroblast-like cells. In contrast, the CM of MSCs inhibited the proliferation of two breast cancer cell lines by arresting the cell cycle at the G0/G1 phase. *In vivo* studies revealed that fluorescence dye-labeled MSCs migrated into tumor tissues. Unexpectedly, single or multiple intravenous injections of MSCs did not affect the latency of breast cancer in TVA- transgenic mice induced by intraductal injection of the RCAS vector encoding polyoma middle-T antigen (PyMT) or Neu oncogenes. Moreover, MSCs had no effect on RCAS-Neu tumor growth in a syngeneic ectopic breast cancer model. While our studies consistently demonstrated the ability of breast cancer cells to profoundly induce MSCs migration, differentiation, and proliferation, the anti-proliferative effect of MSCs on breast tumor cells observed *in vitro* could not be translated into an antitumor activity in *vivo*, probably reflecting the antagonizing or complex effects of MSCs on tumor environment and tumor cells themselves.

## Introduction

Mesenchymal stem cells (MSCs) are bone marrow–derived non-hematopoietic precursor cells that contribute to the maintenance and regeneration of connective tissues through engraftment. MSCs can be obtained from bone marrow aspirates, umbilical cord blood, or adipose tissues and expanded *in vitro*. MSCs are capable of differentiating into bone, cartilage, muscle, fat, and connective tissues throughout the body [1], [2]. MSCs delivered intravenously are able to engraft in tumor tissues and differentiate into carcinoma-associated fibroblast cells. The inflammatory milieu produced by tumors play an important role in developing tumor tropism of MSCs. Growth and angiogenesis factors such as FGF-2 and VEGF, and chemokines such as MCP-1 (monocyte chemoattractant protein-1) and CCL5 (Chemokine C-C motif ligand 5) produced by tumors or their microenvironment can attract migrating MSCs *in vitro* in co-culture experiments [3]–[7]. Induction of chemotaxis and a pro-inflammatory environment induced by radiation therapy can further promote the engraftment of MSCs into subcutaneous tumors formed after transplantation of cells of the 4T1 breast cancer cell line in Balb/c mice [7].

The ability of MSCs to develop tumor tropism has led to the development of MSCs as a novel vehicle to deliver tumoricidel molecules or agents to target tumor cells. For examples, MSCs infected with the vectors expressing IFN-β or TRAIL can suppress the growth of human glioma cell lines in a xenograft model [8]–[10]. MSCs have also been designed as a vehicle for carrying adenovirus to tumor sites [11]–[14]. MSCs infected with adenovirus migrate to tumor tissues and induce an oncolytic anti-tumor activity. Recently, the use of MSCs as a cell-based antitumor therapy has been questioned because of the contradicting reports on the ability of MSCs themselves to suppress or enhance tumor cell proliferation and growth. It appears that the tumor types, the sources of MSCs, e.g. bone marrow-derived versus adipose tissue-derived or umbilical cord-derived MSCs, and mouse models such as syngeneic versus xenogeneic graft are the contributing factors that affect the outcome of MSCs on tumor growth and progression. Therefore, it is highly desirable to investigate the effect of MSCs in a clinically relevant mouse model.

Li and colleagues reported a novel somatic mammary carcinoma model using TVA (the receptor for the sub-group A avian leucosis virus) technology [15], [16]. Transgenic mice with targeted expression of TVA in mammary epithelial cells under the control of the MMTV (murine mammary tumor virus) promoter were generated. Mammary carcinomas become palpable in two weeks in TVA transgenic mice after intraductal injection of RCAS virus (1×10^7^ virions) expressing a viral oncogene, polyoma virus middle T antigen (PyMT) tagged with hemagglutinin (HA). Lowering the number of virions prolonged tumor latency [17]. Unlike the RCAS-PyMT virus, the RCAS-Neu virus induces breast cancer with a long tumor latency (>4 months after viral infection) [15], [16]. In the present study, we have characterized the effect of breast cancer cell lines derived from TVA transgenic mice infected with Neu and PyMT oncogenes on MSC proliferation, migration, and differentiation, and determined whether MSC can affect breast cancer formation induced by these two oncogenes in a somatic mouse model and tumor growth in a syngeneic ectopic breast cancer mouse model.

## Materials and Methods

### Cells

MSCs were isolated from bone marrows of FVB wild-type mice as previously reported [18]. Briefly, the cells from the long bones of FVB mice (6–10 weeks female mice) were isolated by flushing out bone marrows. The cells and aggregates were dispersed and centrifuged at 1500 rpm. The pellets were washed 3 times with Hank's balance salt solution and then seeded in 100-mm tissue culture dishes in DMEM containing low glucose, 10% fetal bovine serum, 35 µg/ml heparin. After incubation at 37°C and 5% CO2 for 24 hours, nonadherent cells were discarded; adherent cells were washed with PBS. Fresh complete isolation medium was added every 3 to 4 days for 4 weeks. To expand MSCs, confluent monolayers of the cells were collected by trypsinization and re-plated in 200-mm dishes. RCAS-Neu and RCAS-PyMT breast cancer cell lines were derived from a breast cancer in TVA-transgenic mice infected with an avian retroviral vector encoding *Neu* or *PyMT* [16]. The genetic backgrounds of these two breast cancer cell lines have not been fully profiled. RCAS-Neu and RCAS-PyMT cells were phenotypically different under a microscope. In addition, we found that RCAS-PyMT cells were unable to form breast cancer when inoculated subcutaneously or by fat pad injection into FVB mice. The 4T1 murine breast cancer cell line and the NIH3T3 fibroblast cell line were purchased from the American Tissue Type Collection (Manassas, VA). The immortalized DF-1 chicken fibroblast cell line, originally obtained from American Tissue Culture Collection (Manassas, VA), was kindly provided by Dr. Y. Li (Baylor College of Medicine, Houston, TX). The use of this cell line has been previously reported [16]. NIH3T3 and DF-1 cells were grown in the complete DMEM medium supplemented with 10% fetal bovine serum, L-glutamine (2 mM), penicillin (100 units/ml) and streptomycin (100 µg/ml).

### Verification of MSC

The cells on passage 3were analyzed by FACS for the lack of the CD45 hematopoietic marker. The mesenchymal lineage was verified by the ability of MSCs to differentiate into adipocytes, osteogenic blasts and chondrocytes. For adipocyte differentiation, confluent MSCs seeded in a 24-well plate were cultured for three weeks with high glucose DMEM (40%), Ham F12 50%, rabbit serum (10%), dexamethasone 100 nM, and insulin 0.5 µg/ml. Same medium was changed every two days. Adipocyte differentiation was analyzed by fixing with formalin and staining with Oil Red O for 10 min.

For osteogenic differentiation, confluent MSCs seeded in a 24-well plate were cultured for three weeks with high glucose DMEM supplemented with 10% fetal bovine serum, β-glycerol-phosphate (10 mM), ascorbic acid (50 µg/ml), dexamethasone 100 nM. Osteogenic differentiation was analyzed by monitoring mineralization by fixing with 10% formalin and staining with Alizarin Red S (2% w/v Alizarin Red S adjusted to pH 4.1 using ammonium hydroxide) for 20 min.

For chondrocyte differentiation, confluent MSCs seeded in a 24-well plate were cultured for three weeks with high glucose DMEM supplemented with 10% fetal bovine serum, ascorbic acid (50 µg/ml), and TGF-β (1 ng/ml). Same medium was changed every two days. Chondrocyte differentiation was analyzed by fixing with 10% formalin and staining with Alician blue (1% w/v Alician blue, pH 1.0 in 0.1 N HCl) for 20 min.

### Cell migration

MSC monolayers grown in T-25-cm2 flasks were detached by using the Cell Dissociation Buffer (Sigma Chemical Co.) and washed twice with the serum-free medium containing 0.1% BSA. The cells (2×10^4^/well) were seeded in the top chamber of the 24-well Transwell inserts. The Transwell inserts were placed in a 24-well companion plate filled with 0.75 ml of conditioned medium of RCAS-Neu, RCAS-PyMT, NIH3T3 cells or control media. After incubation for 24 h, the cells in the inner side of top chamber were removed by wiping with cotton swabs. The cells that migrated through the filter insert to the opposite surface were stained with a Diff-Quik kit (Mercedes Medical, Sarasota, FL). The membrane was sliced out and then mounted onto a hemacytometer and sealed with the mounting media. The cells were counted under a light microscope. The mean ± standard deviation of the cells counted from 5 random fields (20×) was calculated and statistically analyzed using an unpaired student *t* test between different treatments.

### Western blot

MSCs cultured with conditioned media of RCAS-Neu, RCAS-PyMT, or NIH 3T3 cells were harvested and lysed in Nonidet P (NP)-40 lysis buffer (50 mM Tris-HCl (pH 8.0), 150 mM NaCl, 1% NP-40, 5 mM EDTA, 10 µg/ml aprotinin, 10 µg/ml leupeptinin, and 1 mM phenylmethylsulfonyl fluoride). After electrophoresis and transfer to nitrocellulose membranes, vimentin was detected by using a rabbit monoclonal antibody (Cell Signaling Technology, Inc., Danvers, MA), followed by horseradish peroxidase-conjugated goat anti-rabbit IgG and SuperSignal Western Pico enhanced chemiluminoscence substrate (Pierce Chemical Co., Rockford, IL). A monoclonal antibody against β-actin was purchased from Santa Cruz Biotechnology Inc., San Diego, CA.

### MTT assay

MSCs or tumor cell lines were seeded in 96-well plates at the density of 2,000/well. After incubation for 96 hr, cell proliferation was monitored by using a CellTiter 96 non-radioactive cell proliferation assay kit (MTT) (Promegan, Madison, WI) following the manufacturer's instruction.

### BrdU labeling and DNA replication

MSCs were grown in complete DMEM medium with 10% fetal bovine serum in 60-mm dishes. Upon 40% confluence, culture medium was replaced with complete DMEM medium mixed with control medium or conditioned media from two breast cancer cell lines or NIH3T3cells and incubated for 48 hr. After pulse with 10 µM BrdU for 3 hr, MSCs were harvested, denatured with 2N HCl for 5 min at room temperature followed by neutralization with 0.1 M borate buffer (pH 8.5). After washing and blocking with normal mouse serum, the cells were stained with an Alexa Fluor 488-conjugated anti-BrdU monoclonal antibody (BD Bioscience). Alexa Fluor 488-conjugated mouse IgG was included as a control. Cells were immediately analyzed for DNA incorporation in a flow cytometer.

### Immunofluorescence staining

Tumor cell lines grown on coverslips were washed 3 times with cold PBS and fixed with cold methanol at 4°C for 10 min. Coverslips were blocked with 5% normal goat serum for 30 min at room temperature. FAP was detected by a rabbit anti-FAP IgG in IHC staining followed by fluorescein-conjugated goat anti-rabbit IgG. The coverslips were mounted with 50% glycerin in PBS containing anti-fade reagent 1,4-diazabicyclo (2.2.2) octane (25 mg/ml) and 4,6-diamidino-2-phenylindole (0.5 mg/ml; Sigma Chemical Co., St. Louis, MO).

### *In vivo* MSCs identification

This study was carried out in strict accordance with the recommendations in the Guide for the Care and Use of Laboratory Animals of the National Institutes of Health. The protocol was approved by the Institutional Animal Care and Use Committee of Rush University Medical Center (Approval protocol number: 10-032). All surgery was performed under ketamine and zylazine anesthesia, and all efforts were made to minimize suffering. To assess the ability of MSCs migrating into the tumor sites, MSCs were first labeled with fluorescence dye SP-Dil (Molecular Probes) as described previously [18]. Briefly, MSCs grown in dishes were loaded with SP-Dil at a final concentration of 10 µg/ml for 12 hr. The monolayer of MSCs was washed twice with PBS and then incubated in the dye-free complete DMEM medium for 4 hr. The cells were harvested by trypsinizaton and rinsed three times with Hank's buffered salt solution. The cells (2×10^6^ cells/mouse) in single cell suspension were injected into the tail veins of TVA-transgenic mice bearing breast cancer that was induced by infecting with RCAS-PyMT virus three weeks earlier. Mice were sacrificed 4 days later. Tumor tissues were collected and snap-frozen in liquid nitrogen for histological analysis. The sections of cryostat were counterstained with DAPI and examined under a fluorescence microscope. SP-Dil-labeled cells were visualized with red fluorescence.

### *In vivo* tumor induction

TVA-transgenic mice expressing the receptor for an avian retrovirus vector, RCAS, were infected with DF-1 cells transfected with RCAS-PyMT vector or RCAS-Neu by intraductal injection. Mice were treated with MSC (2×10^6^cells/mouse) by tail vein injection of single cell suspension one time as indicated in each experiment. Mice were observed for breast cancer development by palpation. The difference of tumor latency between untreated and MSC-treated groups was statistically analyzed by using the Log-rank test. To determine whether MSCs affect tumor growth, RCAS-Neu cells were co-implanted with MSCs (5×10^5^/gland) at the ratios 1∶0, 1∶0.2, or 1∶1 into the fat pad of FVB female mice. Tumor growth was measured twice weekly for 7 weeks. The difference of tumor growth between three groups was statistically analyzed by using the one-way repeated measure ANOVA. The *p* value of <0.05 was considered statistically significant.

## Results

### Characterization of MSC

**Figure 1:**
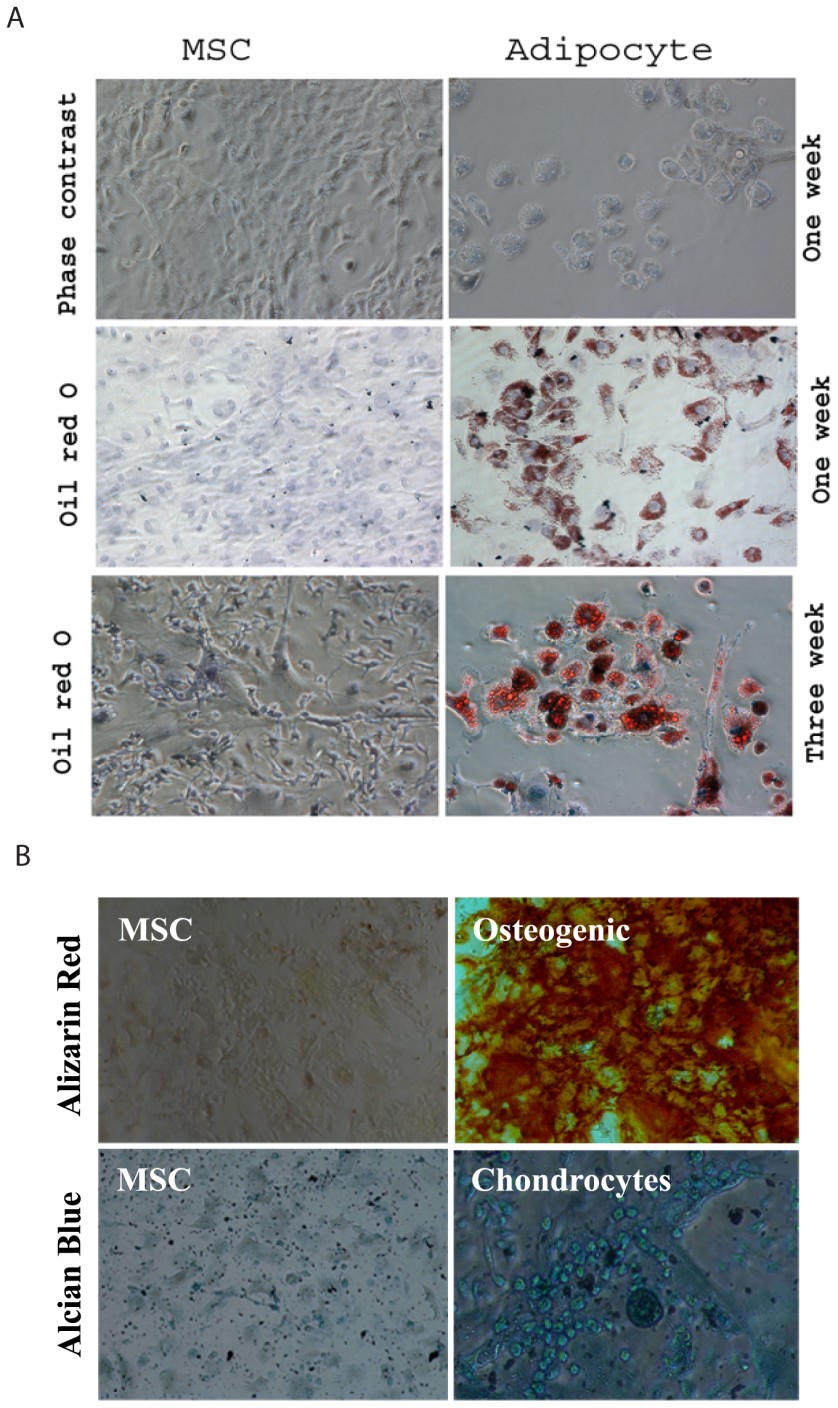
Characterization of MSCs. (A) MSC differentiation into adipocytes. MSCs were grown for 1 or 3 weeks under the conditions for adipocyte differentiation. Cells were fixed and stained with oil red O. Undifferentiated MSCs were used as a negative control. (B). MSC differentiation into osteocytes and chondrocyte. MSCs grown for 3 weeks under the conditions for ostecyte and adipocyte differentiation were fixed and stained with Alizarin Red or Alician Blue. Undifferentiated MSC were used as a negative control.

MSCs were isolated from mouse bone marrow and cultured by continuous growth through adherence to plastic dishes. At passage 3–6, >99% cells are CD45^−^ cells (data not shown). To determine the capacity of MSCs to differentiate, MSCs were cultured in the presence of high glucose, dexamethasone, and rabbit serum to induce MSC differentiation into adipocytes. As shown in Fig. 1B, differentiated adipocytes were stained light red one week and dark red three weeks after the induction of adipocyte differentiation. Fat droplets were accumulated in the cytoplasm of >90% cells, suggesting the differentiation of these BM-derived MSCs into adipocytes. MSCs cultured in the presence of high glucose, β-glycerol-phosphate, ascorbic acid, and dexamethasone for three weeks differentiated into osteocytes, as indicated by dark red staining of Alizarin Red (Fig. 1C). MSCs cultured in the presence of high glucose, ascorbic acid, and TGF-β differentiated into chondrocytes, as indicated by blue color of Alicia Blue-stained cells (Fig. 1C). In contrast, no color staining was observed in undifferentiated MSCs cultured under normal conditions when stained with either Alizarin Red or Alician Blue (Fig. 1C).

### Breast cancer cell lines induce MSC migration

**Figure 2:**
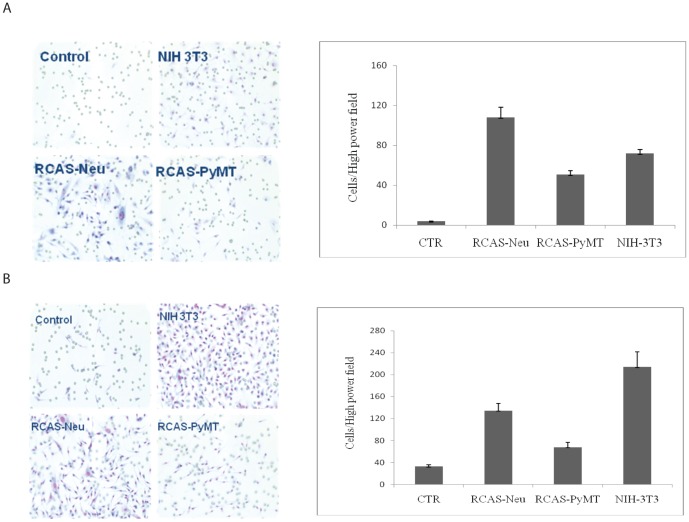
Effect of breast cancer cell lines on MSC migration. MSCs were seeded in the upper wells of Boyden chamber with 0.5-Neu or RCAS-PyMT BC cells or NIH 3T3 cells (0.75 ml each) were placed in the bottom wells (**A**). Instead of conditioned media loaded in the bottom chamber, NIH3T3, RCAS-Neu, or RCAS-PyMT cells (1×10^5^ cells/well) were seeded in the bottom chamber. (**B**). Cells were incubated for 48 hr and stained with Diff-Quick kit for MSCs migrated through the pored membrane.

We next tested whether two breast cancer cell lines, RCAS-Neu and RCAS-PyMT, were able to induce migration of MSCs. Conditioned media (Fig. 2A) from RCAS-Neu, RCAS-PyMT, and NIH 3T3 cells and these three cell lines seeded in the bottom of 24-well plate (Fig. 2B) induced a dramatic increase of the number of MSCs migrated through the inserts of the Boyden chamber. Fig. 2C and D shows the mean ± standard deviation (SD) of the migrating cells counted from 5 fields. The CM of RCAS-Neu cell line was more effective in inducing MSC migration than RCAS-PyMT cell line. Interestingly, NIH 3T3 fibroblast cells or their conditioned media also induced a significant increase of the number of the cells migrating through the membranes of the Boyden chamber (Fig. 2).

### Breast cancer cell lines induce MSC proliferation

**Figure 3:**
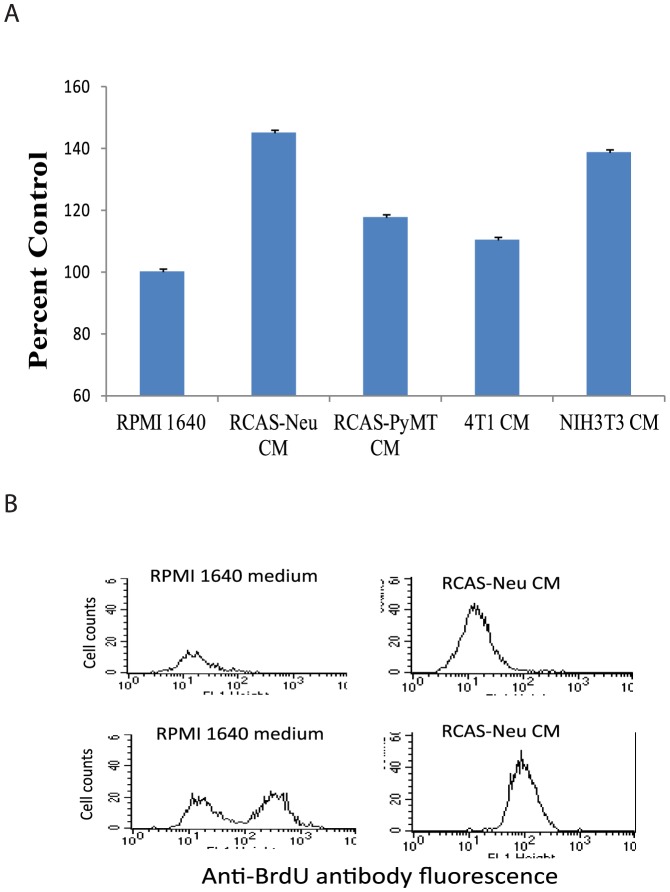
Breast cancer cell lines stimulate MSC proliferation. MSCs were grown in the complete DMEM medium mixed with equal volume of RPMI 1640 media or with conditioned media of RCAS-Neu, RCAS-PyMT or NIH 3T3 cells for 48 hr. Cells were photographed (A) (left panel) and analyzed for cell proliferation by MTT assay (B) (right upper panel). (C) RCAS-Neu CM increases BrdU incorporation in MSCs. MSCs were grown in the complete DMEM medium mixed with equal volume of RPMI 1640 culture media or with conditioned media of RCAS-Neu cells for 48 hr. Cells were labeled with 10 µM BrdU overnight and then analyzed for BrdU incorporation by staining with an Alexa-488-conjugated anti-BrdU antibody. Alexa 488-conjugated mouse IgG was used as an isotype control.

It is possible that increased migration activity of MSCs may result from increased proliferation during 48 hr incubation in the Boyden chamber. We assessed the ability of the conditioned media of these two tumor cell lines to stimulate MSC proliferation. MTT assay revealed that conditioned media of RCAS-Neu significantly increased MSC proliferation by 43%, whereas the conditioned media of RCAS-PyMT did not significantly increase MSC proliferation (*p*<0.05). Interestingly, the conditional media of NIH3T3 but not 4T1 also significantly increased MSC proliferation. BrdU labeling revealed that the conditioned media of RCAS-Neu dramatically increased BrdU incorporation (Fig. 3B).

### Breast cancer cell lines induce MSC differentiation

**Figure 4:**
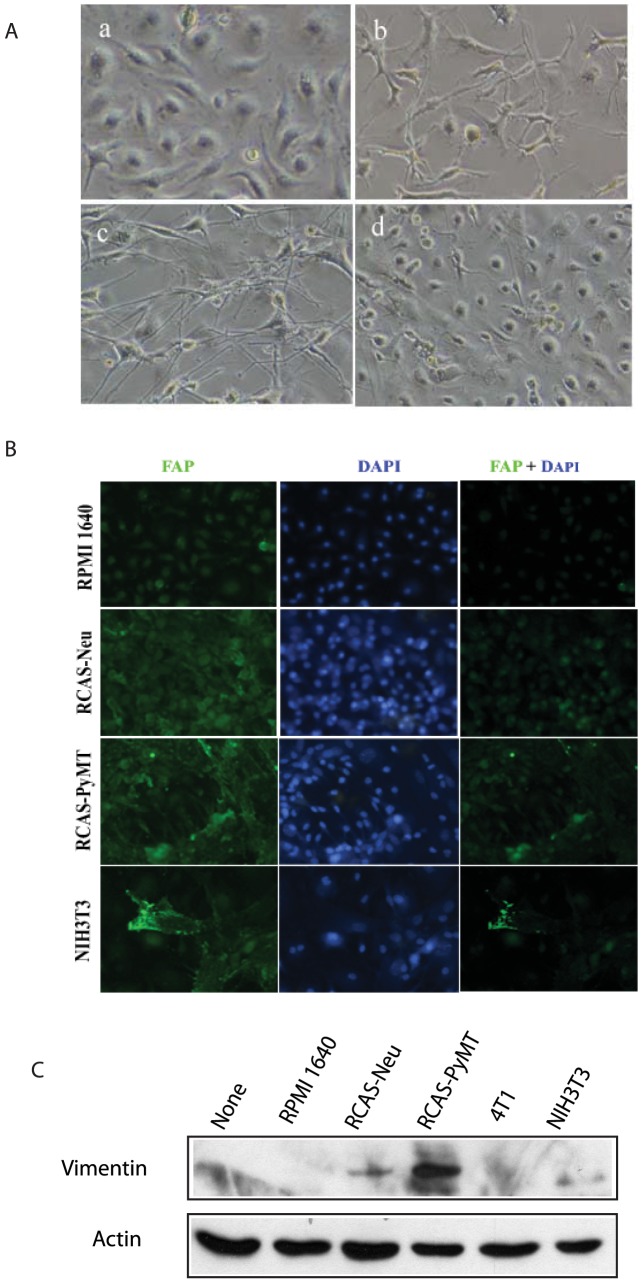
Conditioned media of breast cancer cell lines stimulate MSC differentiation. (A) Morphologic change. MSCs were grown in the complete DMEM medium mixed with equal volume of RPMI 1640 culture media or CM of indicated cell lines. Same culture media were replaced twice weekly. Cells were incubated for 2 wks and monitored for morphological changes. A group of representative photographs taken under a phase-contrast microscopy were shown. (B) Western blot analysis of vimentin. (C) IF staining of vimentin.

MSCs are capable of differentiating into carcinoma-associated fibroblast cells and promoting tumor progression [19]. We tested whether RCAS-Neu and RCAS-PyMT breast tumor cell lines could induce MSC differentiation into fibroblast-like cells. As shown in Fig. 4A, MSCs cultured with the CM of RCAS-Neu and RCAS-PyMT cells for 72 hr undergone a morphological change. The cells became elongated with the appearance of spindle shape. Immunofluorescence staining revealed that these morphologically altered cells from co-culture with the CM of RCAS-Neu and RCAS-PyMT expressed FAP, a marker for fibroblast cells (Fig. 4B). The CM of NIH 3T3 cells induced FAP expression in a far fewer MSCs. Consistently, Western blot revealed the induction of the expression of vimentin, a second marker of fibroblast cells, in MSCs when co-cultured with RCAS-Neu or RCAS-PyMT conditioned media (Fig. 4C).

### Effect of MSCs on breast cancer proliferation

**Figure 5:**
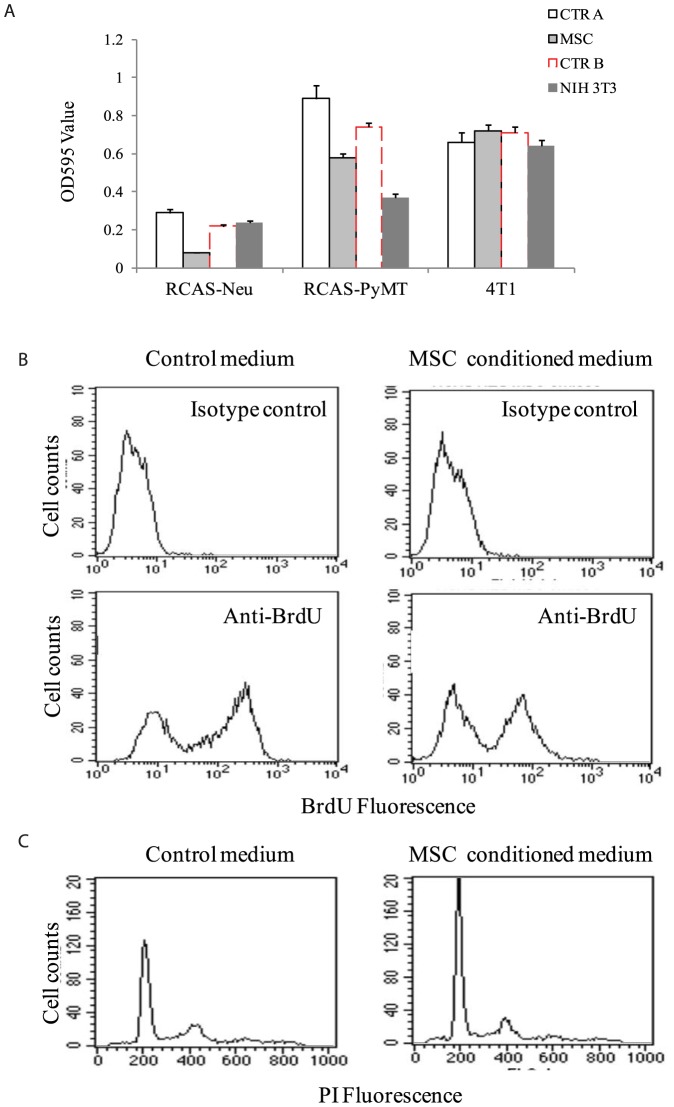
Conditioned medium of MSCs suppresses breast tumor cell proliferation. (A) MTT assay of breast tumor cell proliferation. RCAS-Neu, RCAS-PyMT, and 4T1 cells were cultured in the completed media mixed with equal volume of the conditioned media collected from MSCs or with equal volume of DMEM medium used for culturing MSCs for 48 hr. Cells were analyzed for cell proliferation by MTT assay. (B) RCAS-Neu CM increases BrdU incorporation in MSCs. MSCs were grown in the complete DMEM medium mixed with equal volume of RPMI 1640 culture media or with conditioned media of RCAS-Neu cells for 48 hr. Cells were labeled with 10 µM BrdU overnight and then analyzed for BrdU incorporation by staining with an Alexa-488-conjugated anti-BrdU antibody. Alexa 488-conjugated mouse IgG was used as an isotype control.

We next tested whether the conditioned media of MSCs also affected the proliferation of breast cancer cells. As shown in Fig. 5A, the conditioned medium of MSCs was able to significantly inhibit the proliferation of RCAS-Neu and RCAS-PyMT cells but not 4T1 cells, compared to the control medium used to culture MSCs. In contrast, the conditioned medium of NIH 3T3 cells did not inhibit the proliferation of RCAS-Neu and 4T1 but reduced proliferation of RCAS-PyMT by 50%. We next characterized the nature of MSC CM-mediated anti-proliferative activity toward RCAS-Neu cells, the cell line that was most affected by the conditioned media of MSCs. MSC CM significantly inhibited BrdU incorporation (Fig. 5B) by arresting the cell cycle in the G1 phase (Fig. 5C).

### *In vivo* migration of MSC into tumor tissues

**Figure 6:**
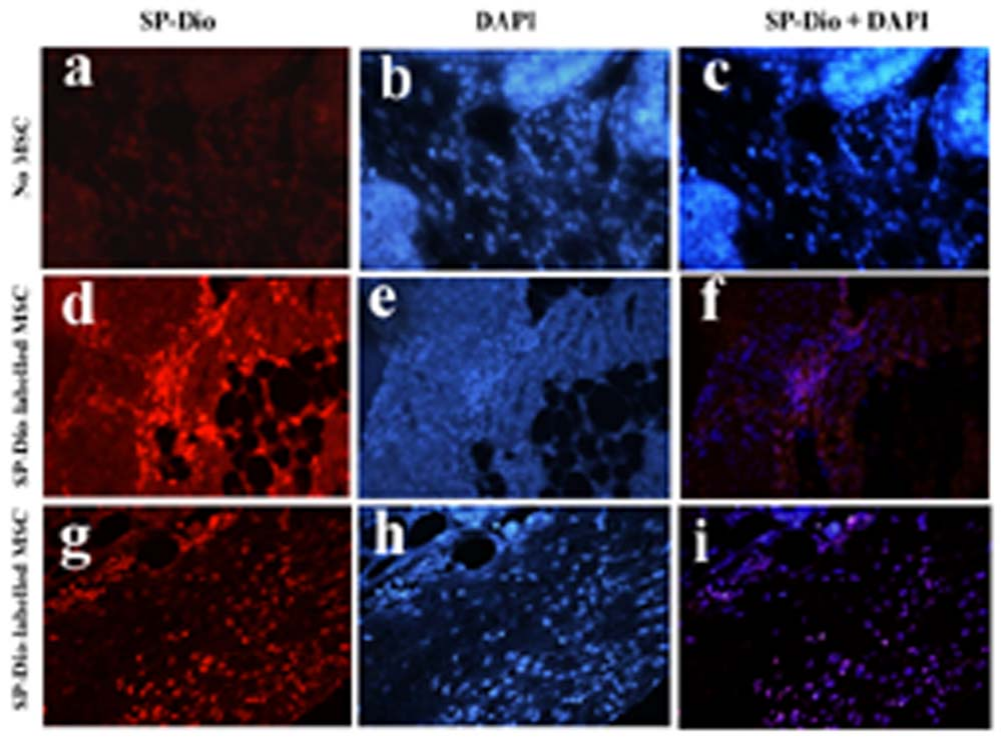
MSCs develop tumor tropism. MSCs labeled with fluorescence dye SP-Dil were injected into the tail veins of TVA-transgenic mice infected with RCAS-PyMT virus three weeks earlier. Four days later, mice were sacrificed and analyzed for the presence of fluorescent dye-labeled cells in the section of tumor tissues under a fluorescence microscope.

We then tested whether MSCs were able to migrate into tumor sites. Mice bearing RCAS-PyMT tumors induced by intraductal injection of RCAS-PyMT-infected DF-1 cells three weeks earlier were sacrificed 3 days after intravenous injection of SP-Dil-labeled MSCs. The sections of tumor tissues were analyzed for the presence of MSCs under a fluorescence microscope. As shown in Fig. 6a, no red fluorescence-labeled cells were present in the sections of tumors from mice receiving unlabeled cells. In contrast, a large number of SP-Dil-labeled cells were present in the stromal tissues between tumor ducts (Fig. 6d) and underneath the tumor capsule (Fig. 6g) in the sections of RCAS-PyMT breast tumors harvested from mice receiving SP-Dio-labeled MSCs.

### Lack of the effects of MSC on breast tumor formation in a somatic breast cancer model

**Figure 7:**
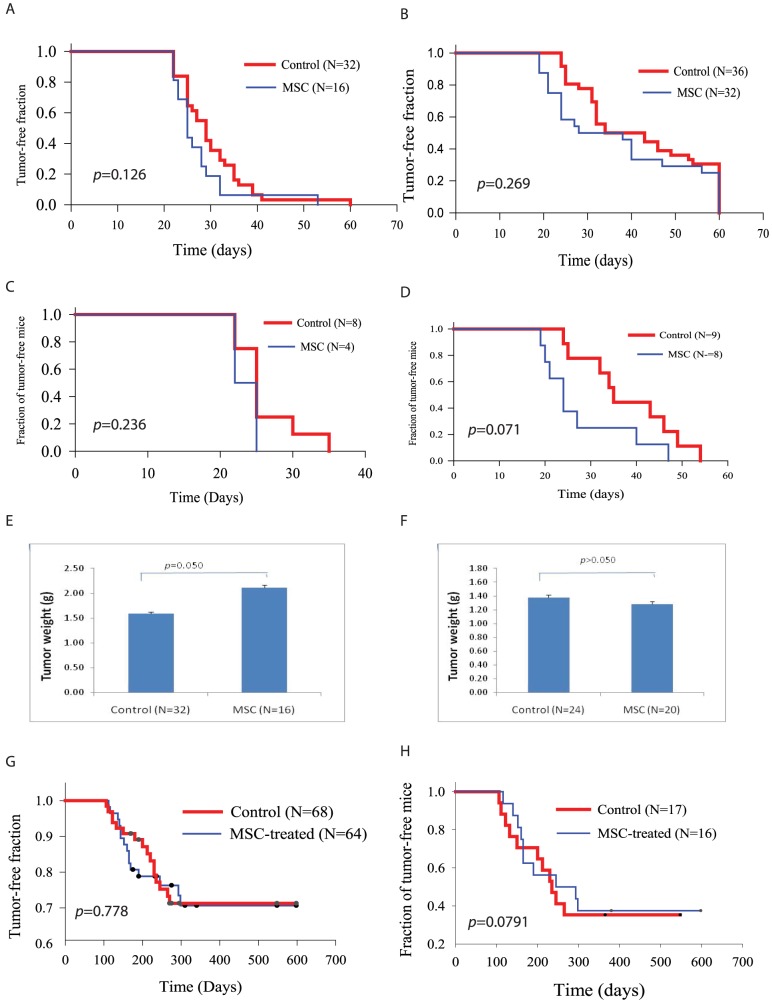
MSCs have no effect on PyMT or Neu oncogene-induced breast cancer. Female TVA transgenic mice (8–10 weeks old) were infected by intraductal injection of RCAS-PyMT virus-infected DF-1 cells (2×10^5^ cells/gland), 4 glands per mouse. Mice were treated with PBS or MSCs [2×10^6^ cells/mouse) once (**A & C**) by i.v. injection one week later or treated with MSCs weekly for 4 weeks (**B & D**)]. Mice were monitored for tumor formation by palpation. Fractions of tumor-free mammary glands infected with RCAS-PyMT DF-1 cells (**A & B**) or tumor-free mice (**C & D**) were shown by Kaplan-Meyer plot. Mice were sacrificed two months later. Tumor nodules were harvested. Tumor weight was recorded and statistically analyzed by using Student *t*-test (**E & F**). (**G & H**) TVA transgenic mice infected with RCAS-Neu virus by intraductal injection of RCAS-Neu virus-infected DF-1 cells (2×10^5^ cells/gland), 4 glands per mouse. Mice were treated three weeks later with PBS or MSCs [2×10^6^ cells/mouse) by i.v. injection biweekly for 8 weeks. Mice were monitored for tumor formation by palpation. Fractions of tumor-free mammary glands (**G**) or tumor-free mice (**H**) infected with RCAS-Neu virus were shown by Kaplan-Meyer plot.

We next tested whether MSCs migrated into breast tumor tissues could affect breast cancer induction. Breast cancer became palpable in TVA transgenic mice 3 weeks after intraductal injection of RCAS-PyMT virus-infected DF1 cells. A Log-rank test revealed that a single injection of MSCs one week after virus infection did not significantly accelerate breast cancer formation induced by PyMT oncogene (Fig. 7A). The mean tumor latency in control mice were 29±5.5 days, versus 27.5±7.5 days in MSC-treated mice. To exclude the possibility that a single MSC injection may not be sufficient to affect tumor development, TVA mice were treated with MSCs weekly for four weeks, starting at one week after tumor induction. As shown in Fig. 7B, the mean tumor latency in control and MSC-treated mice were 42±2.4 and 37±3.3 days respectively. There was no significant difference in the fraction of tumor-free mice (Fig. 7C&D). In addition, we did not find any significant difference in tumor weight (Fig. 7E & F).

We next tested whether MSCs were able to affect Neu oncogene-induced breast cancer formation. Breast cancer became palpable in TVA transgenic mice 3 months after intraductal injection of RCAS-Neu virus-infected DF1 cells. TVA mice were treated with MSCs biweekly for 8 weeks, starting at two week after tumor induction. As shown in Fig. 7G, the mean tumor latency in control and MSC-treated mice were 235±23 and 245±49 days respectively. There was no significant difference in the fraction of tumor-free mice (Fig. 7H) (*p* = 0.0791).

### Lack of the effects of MSCs in stimulating breast tumor growth in a syngeneic tumor model

**Figure 8:**
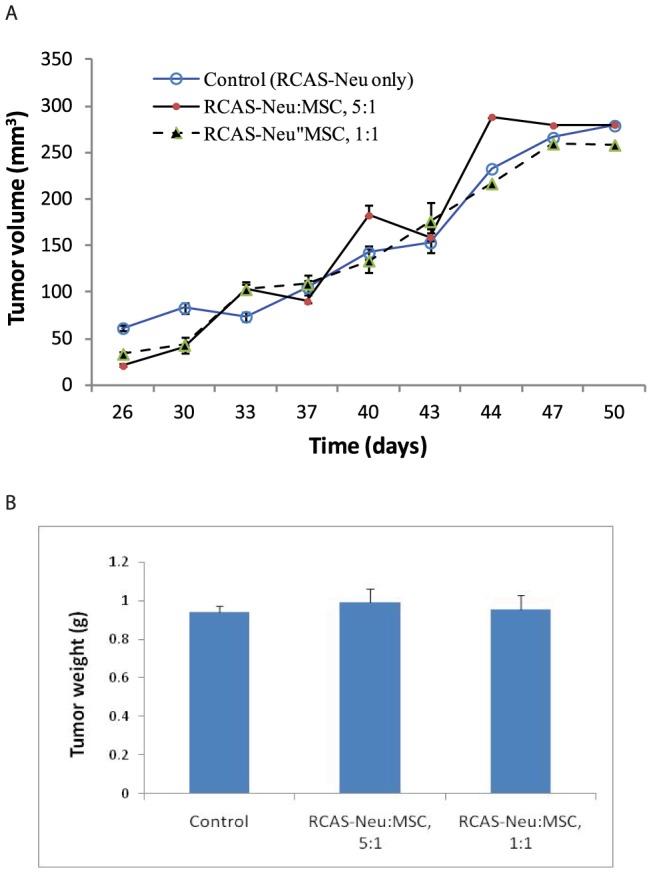
MSCs do not enhance RCAS-Neu tumor growth. RCAS-Neu breast cancer cells (5×10^5^) in a 50 ml volume were mixed with equal volume of PBS or MSCs (1×10^5^ or 5×10^5^ cells.) The mixture (100 µl/mouse) was injected into the fat pad of female FVB mice (6–8 wks of age). Tumor size was measured twice weekly with a calipers.

Finally, we tested whether MSCs co-inoculated with RCAS-Neu tumor cells into the fat pad affected breast cancer formation and growth. Mice receiving RCAS-Neu cells alone were used as a control group. The growth of breast tumors developed from RCAS-Neu tumor cell line co-injected into the fat pad with MSCs at a ratio of 1∶1 or 5∶1 were monitored for 7 weeks. As shown in Fig. 8A, MSC co-injection with either ratio did not affect tumor growth, compared with the control group. There was also no difference in tumor weight between control group and those co-injected with MSCs at the ratio of 5∶1 or 1∶1. RCAS-PyMT cell line was unable to develop breast cancer when injected into the fat pad of FVB mice.

## Discussion

MSCs delivered intravenously or intra-arterially have been shown to engraft within the sites of injured tissue. Tumors are considered to be the “wounds that never heal” [20]. The inflammatory milieu produced by healing wounds and tumors play an important role in developing tumor tropism of MSCs. Our *in vitro* studies demonstrated that two breast cancer cell lines with a different genetic background and their conditioned media were able to induce MSC migration. Breast cancer cells may produce some chemokine factors similar to that produced by fibroblast cells since the conditioned medium of NIH 3T3 cells was also induce MSC migration. Our *in vivo* studies demonstrated that MSCs labeled with the fluorescence dye SP-Dil also migrated into tumor tissues and resided in tumor stroma and underneath the surrounding capsule of breast tumor nodules. These observations suggest that breast cancer is capable of attracting MSCs into tumor tissues.

Development of MSC tropism in tumor tissues has spurred effort to pursue an MSC-based antitumor therapy strategy through delivering anti-cancer agents directly into tumors. However, whether MSCs themselves can suppress or promote tumor growth and metastases remains controversial. Numerous studies showed that MSCs can stimulate tumor growth and progression. Using an orthotopic colonic xenograft model, Shinagawa *et al*. reported that human MSCs admixed with a KM12SM tumor cell line enhances growth and metastases to the liver. Zhu *et al*. [21] reported that MSCs accelerate the growth of the colon cancer cell lines F6 and SW480 in a nude mouse xenograft model. Fierro *et al*. [22] reported that VEGF and IL-6 produced by MSCs can stimulate MCF-7 breast cancer cell proliferation *in vitro*. In addition, MSCs can promote breast tumor metastasis in a xenograft model [23]. The proinflammatory peptide LL-37 can recruit MSCs, leading to the acceleration of ovarian tumor progression [24].

In contrast to these observations, several other studies demonstrated that MSCs are capable of inhibiting the proliferation of various types of tumor cell lines *in vitro* and suppressing tumor growth *in vivo*. For examples, the conditioned media of MSCs isolated from human umbilical cord or Wharton's jelly both can inhibit the proliferation of human breast cancer and other tumor cell lines by arresting the cell cycle in the G2/M phase and induce apoptosis [25]–[27]. Rat umbilical cord stem cells completely abolished rat mammary carcinomas in a syngeneic rat breast cancer model by inhibiting the proliferation of a Mat B III cell line and triggering apoptosis [18]. MSCs isolated from adipose tissues inhibits the proliferation of pancreatic cancer cell lines and a leukemia K562 cell line by arresting the cell cycle in the G0/G1 phase and provoke apoptosis of pancreatic cancer cell lines *in vitro* [28], [29]. MSCs co-inoculated with human tumor cell lines or administered intravenously inhibited the growth of tumor xenograft of hepatoma, lymphoma, Kaposi's sarcoma, pancreatic and breast cancers [25]–[27], [29]–[34]. In the present study, we demonstrated that the conditioned media of bone marrow-derived MSCs were capable of inhibiting the proliferation of two murine breast cancer cell lines that carry the Neu or PyMT oncogene. Unexpectedly, MSCs systemically administered by intravenous injection in a somatic breast cancer model were unable to inhibit the growth or inductionof breast cancer initiated from Neu or PyMT oncogenes. MSC co-inoculation with RCAS-Neu cells was unable to inhibit tumor growth in an ectopic syngeneic breast cancer model. It should be noted that the lack of effect of MSCs on breast tumorigenesis is not likely due to the oncogenes used to induce breast cancer being too strong. While we observed that *PyMT*, a very strong oncogene, had a >90% penetration, *Neu* is relatively weak oncogenic under the condition we used to induce breast cancer, with a 30% penetration and a relatively long relapse. Nevertheless, these observations are consistent with a recent study using TVA system showing that MSCs systemically administered did not inhibit glioma initiation in a somatic glioma mouse model [35].

The anti-proliferative activity of MSCs *in vitro* and their antitumor effects were largely studied using xenograft models in the immune-deficient host with a tumor environment that is different from that in patients or animals with spontaneous breast cancer [36], [37]. For example, angiogenesis in fast growing tumor xenografts is poorly organized, and microvessels in xenograft tissues tends to be leaky [37]. Immune cells infiltrating into tumor tissues are qualitatively and quantitatively different. In particular, NK cells and macrophages in an immune-deficient host are more active and potent. In contrast, both innate and adaptive immunities are active in immune competent host but can be regulated by MSCs [38], [39]. For example, MSCs inhibit dendritic cell maturation and expression of MHC class II as well as co-stimulatory molecules such as CD83. MSCs inhibit the cytotoxicity of NK cells and IFN-γ production. MSCs also suppress T and B cell proliferation and cytokine production, but stimulate regulatory T cell proliferation through the release of HLA-G5. Our present study demonstrated the ability of MSCs to inhibit tumor cell proliferation but lack of the ability to suppress tumor growth *in vivo*. This may suggest that MSCs have a complex role *in vivo*. MSCs could inhibit tumor cell proliferation *in vivo* but at the same time also suppress antitumor immunity, obscuring its anti-proliferative activity observed *in vitro* in cell culture.

Our studies utilized a clinically relevant breast cancer model to investigate the role of MSCs on tumor formation. Neu or PyMT expression is restricted to a few mammary epithelial cells. Clinically, Neu-positive breast cancers in patients originate from a few cells. In contrast, conventional transgenic mice carrying the PyMT or rat Neu proto-oncogene in their genomes are genetically predestined to overexpress Neu or PyMT in all mammary epithelial cells and to develop lethal invasive mammary carcinomas [40]. Our results demonstrated the lack of effect of MSC on breast cancer induction and growth, though multiple administrations of MSCs seem to slightly accelerate PyMT-induced breast cancer (Fig. 7B & D) (statistically insignificant). Lack of effect of MSCs in breast tumor induction and progression suggest that MSCs could be safe for delivering novel antitumor agents for breast cancer treatment.

In summary, our present study demonstrated the ability of breast tumor cells to stimulate the mobility of MSCs *in vitro* and migration into the tumor sites. The conditioned media of two murine breast cancer cell lines were able to stimulate the proliferation of MSCs. In contrast, the conditioned media of MSCs were able to inhibit tumor cell proliferation *in vitro*. However, MSCs co-administered systemically or by co-inoculation were unable to inhibit tumor growth in an ectopic syngeneic breast cancer model or breast tumorigenesisin a somatic breast cancer model, probably reflecting a complex role of MSCs *in vivo*. Overall, our results suggest that MSCs do not promote or suppress tumor growth and development in a clinically relevant mouse model.
